# Classification of caries in third molars on panoramic radiographs using deep learning

**DOI:** 10.1038/s41598-021-92121-2

**Published:** 2021-06-15

**Authors:** Shankeeth Vinayahalingam, Steven Kempers, Lorenzo Limon, Dionne Deibel, Thomas Maal, Marcel Hanisch, Stefaan Bergé, Tong Xi

**Affiliations:** 1 Department of Oral and Maxillofacial Surgery, Radboud University Nijmegen Medical Centre, Postal number 590, P.O. Box 9101, 6500 HB Nijmegen, the Netherlands; 2 Artificial Intelligence, Radboud University, Nijmegen, The Netherlands; 3 Department of Oral and Maxillofacial Surgery, Universitätsklinikum Münster, Münster, Germany; 4 Radboudumc 3D Lab, Radboud University Medical Center, Nijmegen, the Netherlands

**Keywords:** Dental diseases, Oral diseases, Scientific data, Biomedical engineering

## Abstract

The objective of this study is to assess the classification accuracy of dental caries on panoramic radiographs using deep-learning algorithms. A convolutional neural network (CNN) was trained on a reference data set consisted of 400 cropped panoramic images in the classification of carious lesions in mandibular and maxillary third molars, based on the CNN MobileNet V2. For this pilot study, the trained MobileNet V2 was applied on a test set consisting of 100 cropped PR(s). The classification accuracy and the area-under-the-curve (AUC) were calculated. The proposed method achieved an accuracy of 0.87, a sensitivity of 0.86, a specificity of 0.88 and an AUC of 0.90 for the classification of carious lesions of third molars on PR(s). A high accuracy was achieved in caries classification in third molars based on the MobileNet V2 algorithm as presented. This is beneficial for the further development of a deep-learning based automated third molar removal assessment in future.

## Introduction

The removal of third molars is one of the most commonly performed surgical procedures in oral surgery. Recent guidelines recommend the removal of pathologically erupting third molars in order to prevent future complications^1,2^. The second molar is frequently disrupting the eruption path of the third molar, evoking it to only erupt partially or not at all, which can adversely affect periodontal health of the second molar. Impacted or partially erupted third molars are often the cause for various pathology such as pericoronitis, cysts, periodontal disease, damage to the adjacent tooth and carious lesions^3^. The prevalence of carious lesions in third molars is reported to range between 2.5% and 86%^4^.

In the present day, the decision flowchart for third molar removal is made in compliance with national protocols, based on considerations of a wide range of risk factors, including the anatomy-, general health-, age-, dental status, drug history, other specific patient-, surgeon- and financial related factors. The decision whether to remove a third molar or not, can only be made by considering these clinical data with the necessary radiological information, that are present on preoperative panoramic radiographs (PRs)^3^. Occasionally, radiological abnormalities detected on an PR may even require further investigation with a cone-beam computed tomography (CBCT)^5^. Taking the numerous interactions between all those factors into account, it might be challenging to make the correct decision during an average presurgical consultation. An automated decision-making tool for third molar removal may have the potential to aid patients and surgeon to make the right choice^6^. The detection of pathologies associated with third molars on PR is the first step in the automation of M3 removal diagnostics.

In recent years, deep learning models like convolutional neural networks (CNNs) have been used to analyse medical images and to support the diagnostic procedure^7^. In the field of dentistry, CNNs have been applied for the detection and classification of carious lesions on different image modalities such as periapical radiographs^8^, bitewings^9^, near-infrared light transillumination images^10,11^ and clinical photos^12,13^. However, none of the studies have explored automated caries detection and classification on PR(s). The aim of this study is to train a CNN-based deep learning model for the classification of caries on third molars on PR(s) and to assess the diagnostic accuracy.

## Results

**Figure 1:**
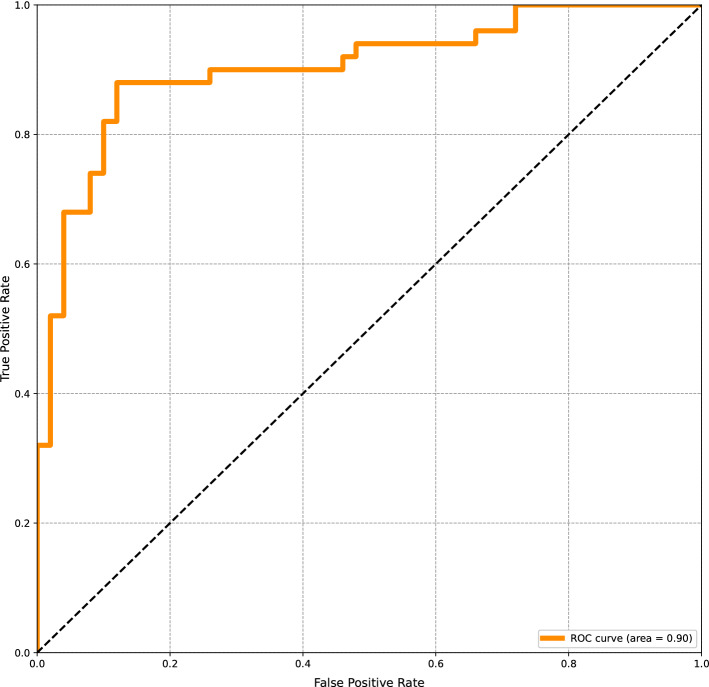
Area-under-the-curve-receiver-operating-characteristics-curve. The ROC curve is created by plotting the true positive rate against the false positive rate at different thresholds.

**Figure 2:**
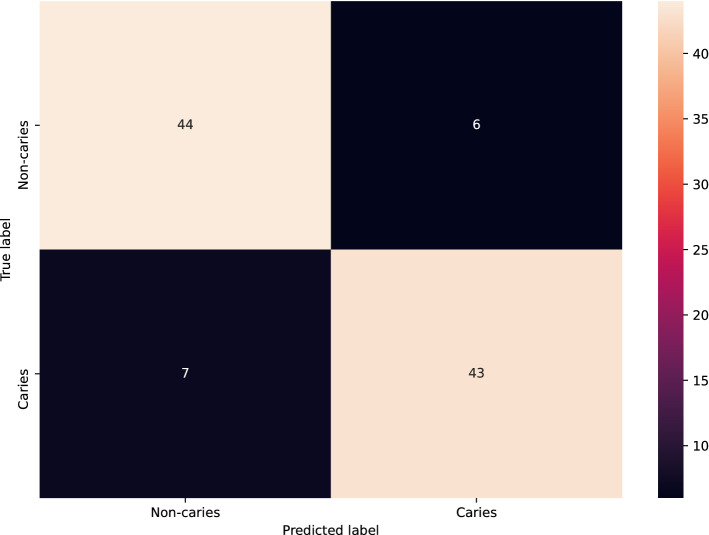
Confusion matrix showing the classification results.

Table 1 summarizes the classification performance of MobileNet V2 on the test set, including the accuracy, positive predictive value, sensitivity, specificity and negative predictive value. The classification accuracy was 87%. The model achieved an AUC of 0.90 (Fig. 1). The confusion matrix is presented in Fig. 2.

**Table 1:** The Accuracy, positive predictive value (PPV), negative predictive value (NPV), F1-score, sensitivity and specificity for the detection of dental caries in third molars on PR(s).

Accuracy	PPV	NPV	F1-score	Sensitivity	Specificity
0.87	0.88	0.86	0.86	0.86	0.88

**Figure 3:**
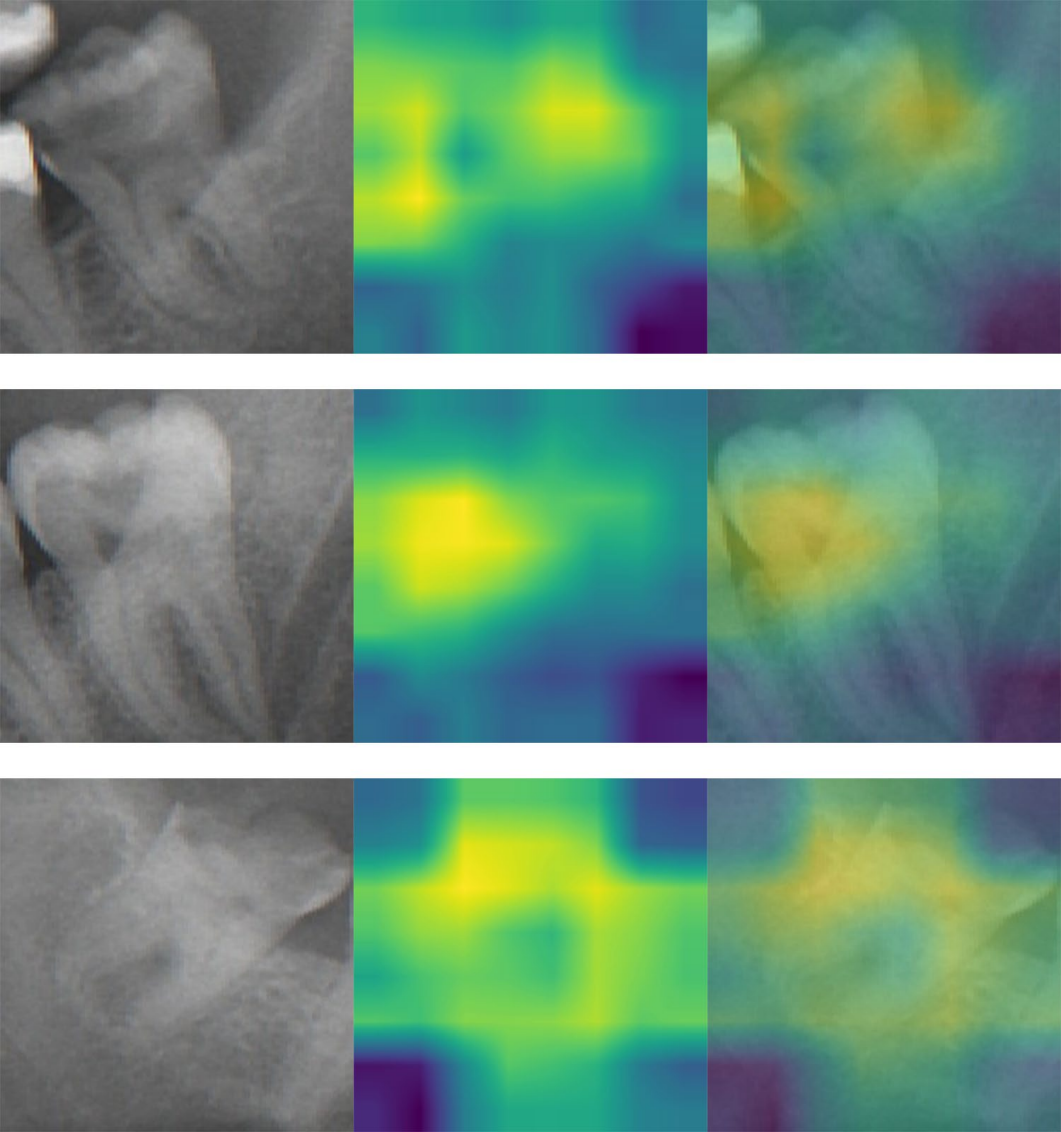
Class activation map for carious third molars. The left column shows the cropped carious M3s. The middle column represents the class activation map. The right column illustrates the overlay.

**Figure 4:**
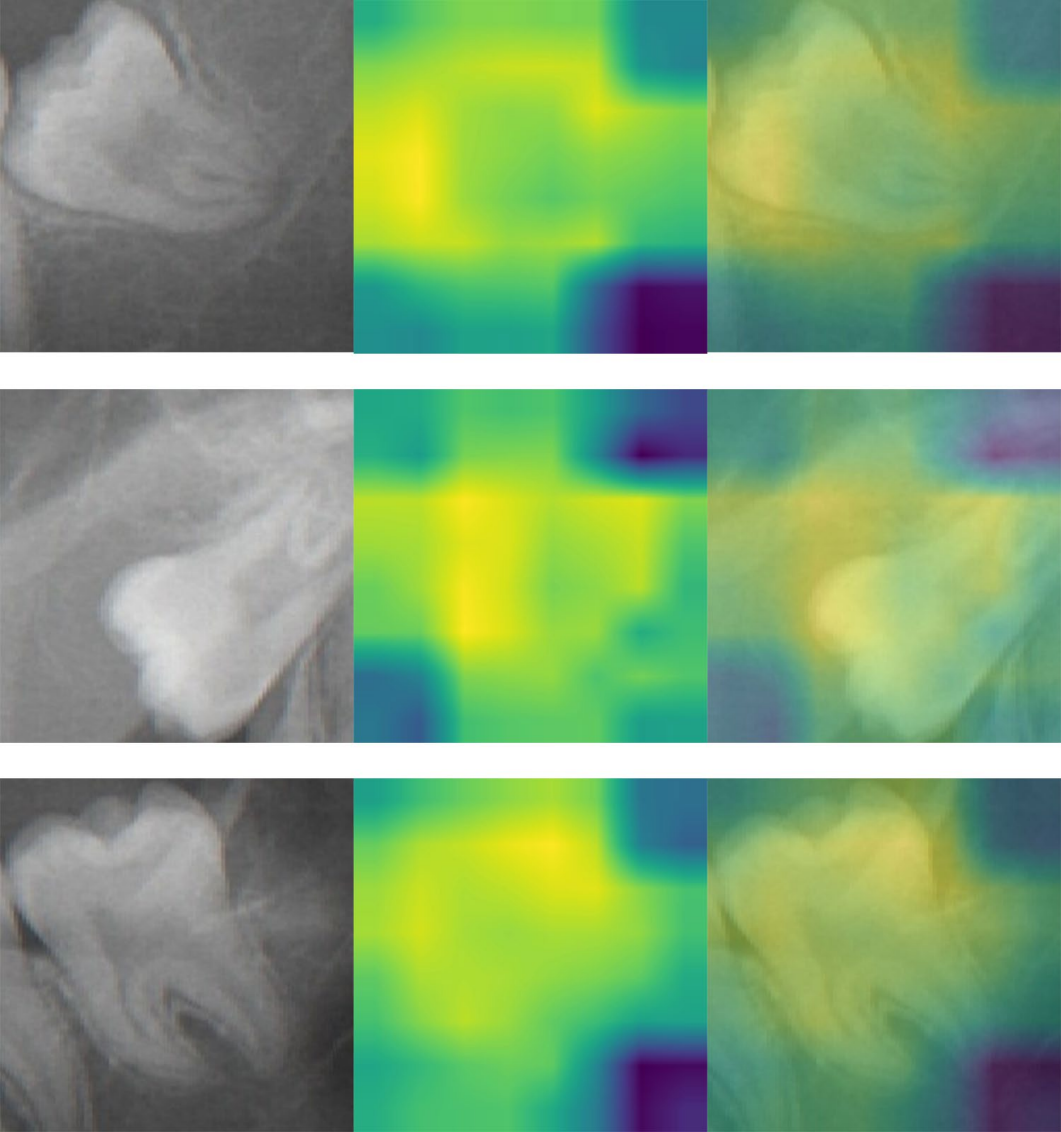
Class activation map for non-carious third molars. The left column shows the cropped non-carious M3s. The middle column represents the class activation map. The right column illustrates the overlay.

The class activation heatmaps (CAM) for carious third molar and non-carious third molar are illustrated in Figs. 3 and 4. These heatmaps visualize the discriminative regions used by the MobileNet V2 to classify the third molar (M3) as carious and non-carious. Optical inspection indicates a broader region of interest in non-carious M3(s). A more centered and focused region of interest is found for carious M3(s).

## Discussion

A daily dilemma in dentistry and oral surgery is to determine whether a third molar should be removed or not. In cases of diseased third molar, where pain or pathology are obvious, there is a general consensus that surgical removal is indicated^3^. Improved diagnostics, e.g. on PR(s), might improve the selection process whether to remove or not. Furthermore it might facilitate the decision process whether an additional CBCT is required to assess the risks and benefits in a more adequate way. A more stringent indication pathway may reduce unnecessary third molar removals, thus reducing the operation-related comorbidity and costs^14^.

This pilot study assesses the capability of a deep learning model (MobileNet V2) to detect carious third molars on PR(s) and is therefore a mosaic stone in the picture of automation of M3 removal diagnostics. Caries classification on third molars using PR(s) is flawed by limited and varying accuracy of individual examiners leading to inconsistent decisions and consequently suboptimal care^9^. The use of deep neural networks might bring us a more reliable, faster and reproducible way of diagnosing pathology, and can therefore reduce the number of unnecessary third molar removals^15^.

In dental radiology, previous studies applied deep learning models for caries detection and classification on different image modalities^8–13,16^. Lee JH et al. applied a pre-trained GoogLeNet Inception v3 CNN network on periapical radiographs achieving accuracies up to 0.89^8^. Casalegno et al. applied U-net with VGG-16 as an encoder, on near-infrared transillumination images (NITS)^10^ with a reported AUC between 0.836 and 0.856. ResNet-18 and ResNext-50 were applied by Schwendicke et al. on NITS^11^. The reported AUC ranged from 0.730 to 0.856 in these studies. Two other studies explored the caries detection on clinical photos using Mask R-CNN with ResNet, reporting an accuracy of 0.870^13^ and a F1-score of 0.889^12^. Finally, U-net with EfficientNet-B5 as an encoder was used to segment caries on bitewings with an accuracy of 0.8^9^. It is important to note that the performance of the deep learning models are highly dependent on the dataset, the hyperparameters, the image modality and the architecture itself^7,16^. As these parameters differed between the studies, a direct comparison of these studies would be misleading.

In this study, an accuracy of 0.87 and an AUC of 0.90 was achieved for caries classification on third molars on PR(s). In comparison, previous studies have stated an AUC of 0.768 for caries detection on PR(s) by clinicians^17^. Several factors are associated with the model performance. Firstly, the use of depthwise separable convolutions and the inverted residual with linear bottleneck reduced the number of parameters and the amount of memory constraint while retaining a high accuracy^18^. These characteristics make the MobileNet V2 less prone to overfitting. Overfitting is a modelling error that occurs when a good fit is achieved on the training data, while the generalization of the model on unseen data is unreliable. Secondly, a histogram equalization was applied on the PR(s) as a pre-processing step. Histogram equalization is a method for adjusting image intensities to enhance the contrast and this can increase the prediction accuracy^19^. Lastly, transfer learning was used to prevent overfitting. Transfer learning is a technique that pre-trains very deep networks on large datasets in order to learn the generic and low-level features in the early layers of the network. By reusing these learned weights on other tasks, the need to re-learn these low-level features in new data sets is eliminated, which greatly reduces the amount of data and timed required to converge such a deep neural network^20^.

A limitation of the present study is that only cropped images of third molars were included. Training and testing the model with cropped premolars, incisors and canines might further increase the robustness and the generalizability to assess all caries on PR(s). Secondly, there are several approaches to detect caries on PR(s) such as object detection, semantic segmentation or instance segmentation. Due to the lack of benchmarks in the field of AI applied in dentistry, comparative studies in the future are required to answer the choice of models more objectively. Thirdly, the clinical and radiological assessment by surgeons is not the gold standard in detection of caries. Histological confirmations of caries and further extension of labeled data are required, to overcome the model’s limits in this present study.

To the best of our knowledge, this is the first publication to rely deep learning using solely PR(s) for caries classification on third molars. Furthermore, class activation maps are generated to increase the interpretability of the model predictions. Considering the encouraging results, future work should reside on the detection of other pathologies associated with third molars such as pericoronitis, periapical lesions, root resorption or cysts. Also, the potential bias in these algorithms with possible risks of limited robustness, generalizability and reproducibility has to be assessed in future studies using external datasets and is a necessary step to further implement deep learning successfully in daily clinical practice. Furthermore, prospective studies are required to evaluate the diagnostic accuracy of deep learning models against clinicians in a clinical setting.

In conclusion, a convolutional neural network (CNN) was developed that achieved a F1 score of 0.86 for caries classification on third molars using panoramic radiographs. This forms a promising foundation for the further development of automatic third molar removal assessment.

## Material and methods

### Data selection

253 preoperative PR(s) of patients who underwent third molar removal were retrospectively selected from the Department of Oral and Maxillofacial Surgery of Radboud University Nijmegen Medical Centre, Netherlands (mean age of 31.7 years, standard deviation of 12.7, age range of 16–80 years, 140 males and 113 females)^6^. The accumulated PR(s) were acquired with a Cranex Novus e device (Soredex, Helsinki, Finland), operated at 90 kV and 10 mA, using a CCD sensor. The inclusion criteria were a minimum age of 16 and the presence of at least one upper or lower third molar (M3). Blurred and incomplete PR(s) were excluded from further analysis. This study has been conducted in accordance with the code of ethics of the world medical association (Declaration of Helsinki). The approval of this study was granted by the Institutional Review Board (Commissie Mensgebonden Onderzoek regio Arnhem-Nijmegen) and informed consent were not required as all image data were anonymized and de-identified prior to analysis (decision no. 2019-5232).

### Data annotation

The present third molars (M3(s)) on PR(s) were classified and labeled as carious M3 and non-carious M3 based on electronic medical records (EMR). Subsequently, a crop of 256 by 256 pixels around the M3 was created. The cropped data consisting of carious M3(s) and non-carious M3(s) were de-identified and anonymized prior to further analysis. All anonymized data were revalidated by two clinicians (SV, MH). In cases of disagreement, the cropped PR(s) were excluded. The final dataset consisted of 250 carious M3(s) and 250 non-carious M3(s).

### The model

**Figure 5:**
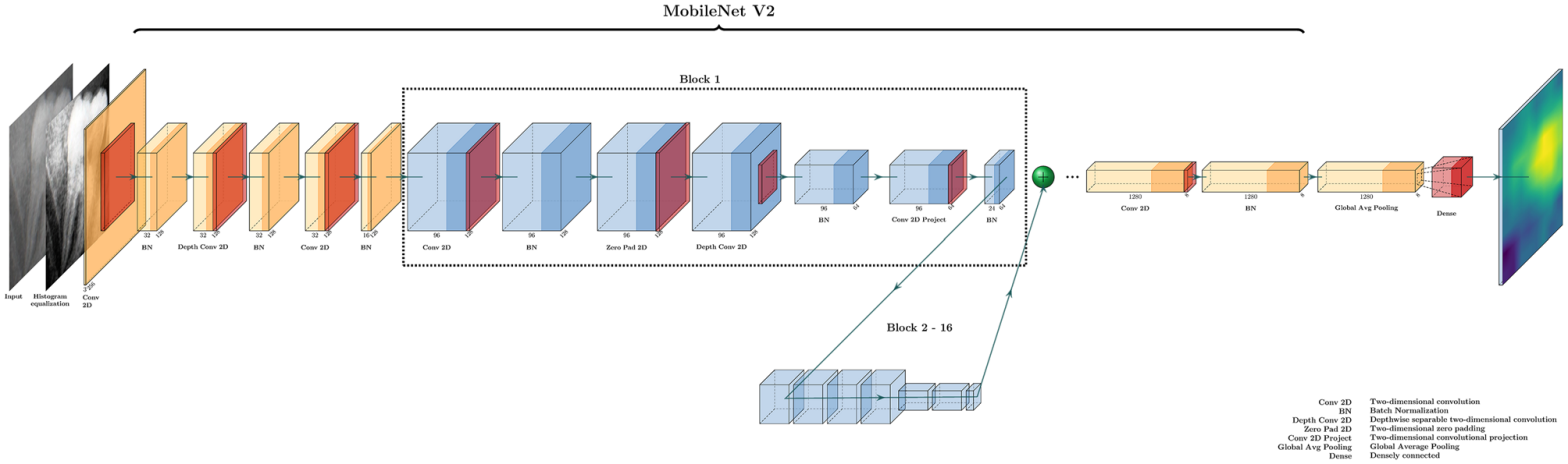
MobileNet V2.

The MobileNet V2 was used in this study. This model is characterized by depthwise separable convolutions and an inverted residual with linear bottleneck. The low-dimensional compressed representation is expanded to a higher dimension with a lightweight depthwise convolution. Subsequently, features are projected back to low-dimensional representation with a linear convolution^18^. The applied model structure is shown in Fig. 5.

### Model training

The total dataset was randomly divided into 3 sets, 320 for training, 80 for validation and 100 for testing. All datasets had an equal class distribution of carious and non-carious third molars. Histogram equalization and data augmentation techniques were employed on the training dataset, in order to improve the model generalization. The MobileNet V2 was pretrained on the 2012 ILSVRC ImageNet dataset^21^. During the training process, hyperparameters and optimization operations were empirically determined, so that a maximum model performance was achieved on the validation set. Subsequently, the best model was used to perform predictions on the test set.

The optimization algorithm employed was the Adam optimizer, at a learning rate of 0.0001, with a batch-size of 32 and batch normalization. The training and optimization process were carried out using the Keras library in the Colaboratory Jupyter Notebook environment^22^.

### Statistical analysis

The diagnostic accuracy for caries classification was assessed based on the true positives (TP), true negatives (TN), false positives (FP) and false negatives (FN). Classification metrics are reported as follows for the test set: accuracy = $\frac{TP+TN}{TP+TN+FP+FN}$, precision = $\frac{TP}{TP+FP}$ (also known as positive predictive value), dice = $\frac{2TP}{2TP+FP+FN}$ (also known as the F1-score), recall = $\frac{TP}{TP+FN}$ (also known as sensitivity), specificity = $\frac{TN}{TN+FP}$, negative predictive value = $\frac{TN}{TN+FN}$. Furthermore, the area-under-the-curve-receiver-operating-characteristics-curve (AUC) and confusion matrix are presented. Gradient-weighted Class Activation Mapping (Grad-CAM), a class-discriminative localization technique was applied, in order to generate visual explanations highlighting the important regions in the cropped image for classifying carious lesions^23^.

## Data Availability

The data used in this study can be made available if needed within the regulation boundaries for data protection.
